# Immunization of preterm infants: current evidence and future strategies to individualized approaches

**DOI:** 10.1007/s00281-022-00957-1

**Published:** 2022-08-03

**Authors:** Mats Ingmar Fortmann, Johannes Dirks, Sybelle Goedicke-Fritz, Johannes Liese, Michael Zemlin, Henner Morbach, Christoph Härtel

**Affiliations:** 1 Department of Pediatrics, University Lübeck, University Hospital Schleswig-Holstein Campus Lübeck, Lübeck, Germany; 2 Department of Pediatrics, University Hospital of Würzburg, Würzburg, Germany; 3 Department of General Pediatrics and Neonatology, Faculty of Medicine, Saarland University Hospital and Saarland University, Homburg, Germany

**Keywords:** Preterm infants, Immunization, Vaccination, Safety, Mechanisms, Resident memory T cells

## Abstract

Preterm infants are at particularly high risk for infectious diseases. As this vulnerability extends beyond the neonatal period into childhood and adolescence, preterm infants benefit greatly from infection-preventive measures such as immunizations. However, there is an ongoing discussion about vaccine safety and efficacy due to preterm infants’ distinct immunological features. A significant proportion of infants remains un- or under-immunized when discharged from primary hospital stay. Educating health care professionals and parents, promoting maternal immunization and evaluating the potential of new vaccination tools are important means to reduce the overall burden from infectious diseases in preterm infants. In this narrative review, we summarize the current knowledge about vaccinations in premature infants. We discuss the specificities of early life immunity and memory function, including the role of polyreactive B cells, restricted B cell receptor diversity and heterologous immunity mediated by a cross-reactive T cell repertoire. Recently, mechanistic studies indicated that tissue-resident memory (Trm) cell populations including T cells, B cells and macrophages are already established in the fetus. Their role in human early life immunity, however, is not yet understood. Tissue-resident memory T cells, for example, are diminished in airway tissues in neonates as compared to older children or adults. Hence, the ability to make specific recall responses after secondary infectious stimulus is hampered, a phenomenon that is transcriptionally regulated by enhanced expression of T-bet. Furthermore, the microbiome establishment is a dominant factor to shape resident immunity at mucosal surfaces, but it is often disturbed in the context of preterm birth. The proposed function of Trm T cells to remember benign interactions with the microbiome might therefore be reduced which would contribute to an increased risk for sustained inflammation. An improved understanding of Trm interactions may determine novel targets of vaccination, e.g., modulation of T-bet responses and facilitate more individualized approaches to protect preterm babies in the future.

**Supplementary Information:**

The online version contains supplementary material available at 10.1007/s00281-022-00957-1.

## Introduction

Preterm birth is a relevant health issue and affects 6–12% of newborn infants worldwide. Preterm infants have an increased risk to suffer from infections during the neonatal period but also carry 1.5–fourfold increased risk for re-hospitalization due to infections during infancy, childhood and adolescence [1]. This is mainly attributed to a combination of physiological constraints, including antigen-naïve immunological phenotype but also gestational age-related aspects of barrier immaturity and small anatomy, reduced vertical transfer of protective maternal antibodies and the high exposure to immunological challenges through invasive measures. In contrast to previous paradigms of a “deficient” immune system, the neonatal immune system should be regarded as distinct on the purpose of adaptation to the outside world. In the context of preterm birth, the delicate balance between “tolerance” (permissive colonization) and immune defense against a variety of microbial antigens is confronted with greater demands than in term infants. Disturbance of this balance during a critical time period of development may result in significant morbidity early and later in life, i.e., enhanced risk for chronic lung disease (CLD) and asthma, neuropsychiatric and cardiovascular disorders [2]. To prevent these long-term consequences after infection, a deeper insight into mediating potentially malleable processes such as (i) sustained, less controlled inflammation, (ii) microbiota distortion and (iii) dysregulation of immunometabolism is needed [3].

Vaccinations are affordable preventive measures to reduce the burden of infections in infants and to save millions of lives [4]. Preterm infants are at particular risk for vaccine preventable diseases, i.e., 2.5–fivefold relative risk to suffer from severe rotavirus infection, invasive pneumococcal disease or pertussis [5]. However, the historical skepticism to vaccinate most susceptible preterm infants has hampered schedule-based vaccinations according to chronological age in many neonatal intensive care units (NICUs), special care nurseries and outpatient settings. For example, approximately 50% of preterm infants in the USA are not vaccinated on time [6]. Particular reasons for this delay include assumptions on (i) “deficient” vaccine-induced immune responses, (ii) tipping the balance toward pro-inflammation through vaccines during vulnerable periods of disease trajectories (e.g., infection risk, developmental window for neurological sequelae, chronic lung disease and retinopathy) or (iii) potential harm of immunizations in the timeframe of expected interventions (i.e., surgery). The observational data from large preterm cohorts such as the German Neonatal Network (GNN) indicate that timing of vaccinations based on recommended schedule does not aggravate the risk for prematurity-related diseases such as retinopathy of prematurity (ROP) and CLD in very-low-birth-weight infants (VLBWI) [7]. Despite evidence about the tolerability, safety, immunogenicity and efficacy of immunizations, the real-life implementation of schedule-based vaccinations by healthcare professionals and parents still remains a challenge.

Recent investigations note that the protective effects of timely vaccinations are not only directed against vaccine-preventable diseases but may also induce accelerated priming of innate immune responses [8]. In the context of preterm infants, this “immune training” reduces the risk for bronchitis during infancy [7]. The concept of “trained immunity” or “innate immune memory” was previously derived from animal models demonstrating a prolonged state of “cell mediated acquired resistance” to multiple secondary pathogens after exposure to primary microbial antigens [9]. A crucial mechanism of trained immunity is the functional upregulation of innate immune cells through epigenetic and metabolic reprogramming. In addition, non-specific priming of natural killer cells, gene silencing of inflammatory pathways by chromatin alterations, programming of myeloid cells, chromatin modifications in monocytes or effects on pro- and anti-inflammatory cytokine responses may contribute to innate immune memory functions [10]. It has been proposed that the early postnatal period preceding discharge may represent the time window during which preterm infants are most receptive to trained immunity effects [8; 11].

Another significant aspect of protecting preterm infants is full immunization of the (becoming) mother (e.g., pertussis, tetanus, SARS-CoV-2, influenza vaccination during pregnancy), close contacts and family members of preterm infants [12].

In this narrative review, we will discuss the importance and challenges of immunization of preterm infants including mechanisms of vaccine responses with regard to specificities of the preterm immune system and future concepts to optimize immunization strategies.

## Safety, tolerability and efficacy of vaccines administered to preterm infants

The protective effect of current vaccines administered in early life is based on the production of neutralizing antibodies. To achieve these memory effects as early as possible, preterm infants should be immunized at the same chronological age as their term counterparts. The mechanisms affecting the strength of vaccination responses in preterm infants are complex and mainly influenced by host factors (genetic background, gestational age and chronological age), environmental aspects including immune-microbiota interaction, maternal antibody levels and prior antibody exposure [13]. Furthermore, vaccine aspects such as timing, route, type of antigen (protein, polysaccharide, live-attenuated viruses) and adjuvants are crucial. We hereby summarize the current evidence on vaccinations administered in the first (most vulnerable) months of life of preterm infants (Table 1, supplement 1). Specifically:

**Table 1:** Safety and immunogenicity of immunizations in preterm infants (PI). AE, adverse event; GA, gestational age; BW, birth weight; CRI, cardio-respiratory instability; CDL, Chronic lung disease; ELGAN, extremely-low-gestational-age neonate; FTI, full-term infant; HBIG, Hepatitis B Immune Globuline; LBWI, low-birth-weight infant; PI, preterm infant; VLBWI, very-low-birth-weight infant; RCT randomized controlled trial; SGA, small-for-gestational-age; TB, tuberculosis; WHO, World Health Organization [80]; AGH, Australian Government – Department of Health [81]; GOC, Government of Canada – Department of Health [82]; AAP, American Academy of Pediatrics [17, 83, 84]

Immunization	Safety	Immunogenicity / efficacy	International recommendations
Rotavirus vaccine (Oral administration) Attenuated human or bovine live vaccine Rotarix® (RV1) RotaTeq® (RV5)	•safe, well tolerated, and effective in PIs [1–7] •rare AEs: intussusception, CRI, viral shedding of vaccine-virus strains [1, 5, 8] RCTs: [2, 9, 10]	•85,7% anti-rotavirus IgA-seroconversion rate [10] •decreased number of post-discharge hospitalizations [1, 4, 5] •prevention of 70% of rotavirus gastroenteritis cases [4] RCTs: [2, 9, 10]	•WHO: PI can be immunized at their chronological age •AGH: WHO recommendations also apply for hospitalized infants who are medically stable •GOC: recommended for healthy PIs
Hepatitis B vaccine (intramuscular) Monovalent for birth-dose i.e., Engerix B® Combination vaccines with DTP, Hib, IPV or Hep A	•no AEs monitored for hepatitis B vaccines [11, 12] •post-exposure prophylaxis well tolerated [12] •exclusively mild AEs for combination vaccines [13, 14] no RCTs	•poorer immune response in PI [15–19] •a booster dose at the age of 1 year ensures comparable levels antibody titers to former term infants no RCTs	•WHO: birth dose can be given to LBWI and PIs but should not count as part of the primary 3-dose series •AAP/GOC: PI < 2000 g: immunization at one month of age or at discharge •HBsAg-status of mother positive/unclear: birth dose + HBIG within 12 h after birth
BCG vaccine (intradermal or percutaneous) Live-attenuated vaccine	•safe administration at birth or delayed in clinically stable PIs [20–2325] •rare AE: non-suppurative lymphadenopathy [24] •limited data on ELGANs RCTs: [24, 25, 27]	•reduces risk of TB by up to 83% and induces protection for 10 years [28] •overall immunogenicity of > 95% (PI > 31 weeks) [20, 29] •reduces mortality in high-risk regions [30, 31] RCTs: [24, 32, 33]	•WHO: Moderate-to-late PIs who are healthy and clinically stable can receive BCG vaccination at birth, or at the latest, upon discharge •GOC: any time > 31 weeks of gestation if indicated
Hexavalent vaccine (intramuscular) DTPa-HepB- IPB-Hib vaccine Diphteria toxoid Tetanus toxoid Bordetella p. Ag (incl. toxoid) Hep. B surface Ag Inact. Poliovirus Hib polysaccharide	•CRI as a non-specific self-limiting stress reaction especially in most vulnerable infants [14, 34–37] but no difference in CRI-rates in RCT between DTaP exposed and unexposed PIs [38–40] •post-immunization fever: 1.4%—33% [13, 41–43] RCTs: [38, 44]	•excellent immunogenicity of most hexavalent components across all GA- and BW- groups [14, 45] •inconclusive reports for pertussis and Hib vaccines [45–48] •Lower response of PIs to certain polio serotypes [14, 49-51], Hib [52–54] and HBV [15–17, 19, 55] no RCTs	•AAP/AGH/WHO/GOC: immunization of PIs according to chronological age without correction for prematurity •AGH: extra dose of Hib for ELGANs or VLBWI at 6 months of age depending on the vaccine used
Pneumococcal vaccine (intramuscular) Conjugate vaccines (i.e., PCV13) Polysaccharide vaccines (i.e., PPSV23)	•safe and well tolerated by PI [56, 57] •comparable rate of AEs after immunization (compared to FTI) [58, 59] •no serious, vaccine-related AEs reported RCTs: [56]	•inconclusive results: GA-dependent specific IgG antibody titers [61] •reduced but protective responses to certain PCV serotypes [62,57,63,64 (importance of a booster dose [62]) •significant reduction in IPD in birth cohorts of PI [65] and up to 100% efficacy for PI and LBWI [59, 60] RCTs: [56]	•AGH/GOC: PIs < 28 weeks (or high risk of IPD) should receive 4 doses of *PCV13 (*and 2 doses of PPSV23 later) •AAP: medically stable PI and LBW should receive PCV beginning at 2 months of age
Meningococcal vaccine (intramuscular) Recombinant vaccines Group B: 4C-MenB Group C: MCC	•safe, generally well tolerated [66, 67] •no serious AEs reported no RCTs	•immunogenic in PIs [53, 66, 68]; similar immunogenicity between PI and FTI (> 99%) [66, 68] •high importance of booster dose to ensure long-term protection [66, 68] no RCTs	•vaccination with meningococcal vaccines (group B and C) is not uniformly recommended by international institutions for PIs explicitly
RSV (intramuscular) Monoclonal anti-respiratory syncytial virus (RSV) antibody (Palivizumab)	•safe and well tolerated in PIs via intramuscular [69–71] and intravenous [72] injection •6.9% mild AEs [71] RCTs: IMpact study [70, 72]	•55% relative reduction for hospital re-admission in PIs [70] •no difference for length of hospital stay, duration of ventilation and mortality [70] •inconclusive efficacy data for late PIs > 33 weeks [73, 74] RCTs: IMpact study [70]	•different international risk-based approaches: •GOC: recommended for PIs who are 6 months of chronological age or younger at the start of the RSV season •AAP: PIs < 29 weeks or < 32 weeks with CLD
Influenza (intramuscular) Inactivated influenza vaccine (IIV)	•limited safety-data in PIs •9% mild AEs [75] •AEs comparable between PI and FTI [76] •serious AEs are very rare [75–77] no RCTs	•comparable immunogenicity in PIs and FTI > 6 months of age [78, 79] •reduced immunogenicity, especially in infants < 6 months [75] •efficacy uncertain (very limited data for PIs) no RCTs	•AGH/AAP: PI should receive 2 doses, at least 4 weeks apart, starting at ≥ 6 months of age and as soon as possible before/during influenza season. One dose every year after that

## Rotavirus vaccines prevent 70% of rotavirus infection-related hospitalizations in preterm infants

Rotavirus (RV) vaccinations are based on live vaccines. Their safety, tolerability and efficacy have been demonstrated for preterm infants in multiple studies including large randomized controlled trials (RCT). The Rotavirus Efficacy and Safety Trial (REST) enrolled 68.038 infants including 2.074 preterm infants receiving either three oral doses of live pentavalent human-bovine (WC3 strain) or placebo. No differences were found between verum and placebo regarding adverse events (including the rare event of intussusception; 1–2/100.000) [14]. Notably, a prolonged diarrhea after rotavirus vaccination maybe the first clinical sign pointing to the diagnosis of severe combined immunodeficiency [15]. Despite immunological immaturity, prolonged diarrhea has not been documented in a remarkable fashion in extremely preterm infants. There is, however, evidence of viral shedding of vaccine strains after immunization, although nosocomial infections with vaccine strains have not been reported to follow a severe course [16]. Most units therefore prefer to vaccinate preterm infants just before hospital discharge in order to protect other potentially immunocompromised patients in the NICU. In extremely preterm infants, this strategy is often incompatible with the recommended timeframe of rotavirus vaccination at 6–12 weeks postnatal age. In this context, vaccination should not be postponed if single family rooms/isolation options exist.

As efficacy measure, a significant decrease in hospitalizations of vaccinated preterm infants under the age of 3 years by 70% and IgA seroconversion rates of > 85% even in preterm infants < 28 weeks of gestation have been documented [17]. However, detailed data on RV5-immunogenicity are scarce. The World Health Organization (WHO) recommends routine vaccination against rotavirus, especially in countries with high mortality from diarrheal illness and for certain risk groups such as preterm infants.

## Multivalent combination vaccines should be administered at chronological age

Multivalent combination vaccines are frequently administered to preterm infants and have several benefits including reduced burden of injection procedures and improved adherence to recommendations. Multiple studies have reported on safety following combination vaccines of DTaP-IPV-Hib-HepB (diphtheria, tetanus, acellular pertussis–inactivated poliovirus–haemophilus influenzae b–hepatitis B) which is frequently co-administered with pneumococcal vaccines [18]. HepB vaccination is either recommended to be administered immediately after birth (WHO, US) or included in the multivalent vaccination. The latter has been proven to be well tolerated by preterm infants, while data on separately administered HepB vaccines are very limited. However, the most relevant adverse events in preterm infants are cardiorespiratory instabilities in timely association with vaccination (particularly first-time immunization), although high-quality evidence is scarce [19]. Events of bradycardia (or apnea) represent rather non-specific procedural responses than vaccine-specific side effects and are most frequent in infants with lowest gestational age [20]. For DTaP, the only prospective, double-blinded, randomized, multicenter trial on post-vaccination cardiorespiratory instabilities in 93 extremely preterm babies did not find differences in immunized compared to non-immunized infants in the first 48 h after immunization [21]. Hence, monitoring for cardiorespiratory events in the 48 to 72 h after vaccination is now recommended for infants at high risk, i.e., < 29 weeks of gestation, < 1000 g birth weight or specific patterns of chronic morbidities. Infants may develop fever after hexavalent vaccination. As a consequence, preterm infants in hospital will be more often exposed to sepsis workup and antibiotic treatment than their term, outpatient counterparts [18]. With regard to efficacy, preterm infants are able to mount protective ranges of antibody responses to hexavalent components of the DTaP-IPV-Hib-HepB while reduced antibody titres have been reported for certain IPV serotypes (serotype III) [22], the acellular pertussis toxin [23] and HepB [24]. Lower geometric mean antibody titres (GMTs) for polio serotype III (and no differences with regard to serotype I and II) were observed in preterm infants as compared to full-term infants throughout infancy [25] and at seven years [26] even after a booster-dose. Study results for Hib are inconclusive. Preterm infants < 32 weeks of gestation reached protective levels of Hib antibodies in 55%–68% after a 2, 3, 4-month-schedule of hexavalent immunization (vs. 80% in term infants) [27]. In a recent study including preterm infants < 28 weeks of gestation, the primary series of immunizations resulted in Hib protective antibodies in 40% of preterm infants [28]. After a booster dose, 81.7% of a preterm group aged less than 28 weeks achieved protective antibody levels. Similarly, protective antibody responses were observed against tetanus and diphtheria in preterm infants, while lower antibody levels were found for Hib and HepB [29]. In this study, however, preterm infants had a diminished immunological memory to pertussis. Moreover, cell-mediated recall responses of preterm infant leukocytes to short hepatitis B exposure were reduced as compared to term infants and dependent on pre-existing antibody levels. Gestational and chronological age might be key factors of reduced immunogenicity to some vaccines, e.g., HepB, or vaccine components [30], and preterm infants may require an extra dose or early booster vaccination [28, 29].

## Streptococcus pneumoniae (Pneumococci) vaccination has significantly reduced the burden of invasive pneumococcal disease (IPD) in preterm infants

Pneumococcal conjugate vaccines (PCV; PCV-7, PCV10 and PCV-13) are well tolerated in preterm infants [30], while vaccine-related serious events were not reported for PCV10 [31] and PCV7 [32]. The efficacy of the PCV immunization in preterm and low birth weight infants was measured with up to 100% [33], and a significant decrease in IPD in preterm (and term) infants after introduction of PCV routine immunization was observed by German surveillance data [34]. Primary series of vaccination in highly preterm infants may result in insufficient levels of protective antibodies, i.e., pneumococcal serotypes 4, 6B, 18C, and 23F between 45.8% and 75.1%, while a booster dose showed high immunogenicity [28].

## Vaccinations against Neisseria meningitidis (Meningococci)—the need for controlled data in preterm infant cohorts

Vaccines against meningococcal diseases caused by serotypes B (MenB) and C (MenC) are well tolerated in extremely preterm infants [35]. A trend toward lower GMTs in preterm infants was observed after primary immunization, while a booster dose induced comparable antibody levels in preterm and term infants at the age of one year [36]. Apart from mild local reactions, serious vaccine-related events were not reported for MenB nor for MenC immunizations, while controlled studies are lacking. Schedules with early immunization recommending a first dose at the age of 8 weeks aim at providing protection for the first peak of meningococcal disease at 5 months of age [35].

## Vaccination of preterm infants against Tuberculosis (Bacillus Calmette–Guerin, BCG) induces trained-immunity effects

The live-attenuated BCG vaccine is one of the most widely used vaccines across the world. Preterm infants are either immunized at birth or at corrected age of 34 weeks gestation. BCG immunization to newborns significantly reduces the risk of tuberculosis by 50% to 83% [37] and induces protection for up to 10 years. Efficient immunogenicity—defined as positive Mantoux test, scar formation, IFN-ɤ rise at 6 months of age—is reported in up to 98% of immunized preterm infants at 31–33 weeks of gestation [38]

BCG immunization is probably the best-known example for a vaccination-related innate immune memory effect [39]. It protects not only against tuberculosis but also improves defense mechanisms against respiratory tract infections and neonatal sepsis [40]. In large scale studies, resistance through non-pathogen-specific (“agnostic”) effects, BCG vaccination given at birth can reduce mortality in 38% (BCG alone) or 40% (BCG + oral polio vaccine), respectively [41, 42]. Experimental models also indicate trained immunity effects of BCG vaccination against malaria and yellow fever. [43, 44].

Safety of BCG vaccination has been mainly demonstrated in cohorts of moderate preterm infants [38]. Non-suppurative lymphadenopathy was the only complication, which was observed in 3.4% of the patients and in < 1% in an RCT from Guinea-Bissau [45]. According to the WHO estimation, severe local reactions occur in 1/ 1.000–10.000 doses, while severe systemic events are very rare (1/ 230.000–640.000 vaccination doses). As data on safety and immunogenicity of BSG vaccines are limited in infants < 30 weeks, the WHO recommends to determine the optimal time point of BCG immunization in this vulnerable group on an individual basis.

## Age-dependent immunogenicity limits protection of the most susceptible infants against influenza

Infants born preterm are at increased risk for hospital (re-)admission due to complicated influenza courses, particularly if they suffer from chronic lung disease [46]. Vaccination is recommended as early as 6 months of chronological age, since immunogenicity might be hampered at an earlier age due to the interference with maternal antibodies. This limits the protective potential during a period of high susceptibility [47]. Immunological responses of 6–17-month-old preterm infants to influenza vaccines are comparable to those with full-term infants [48], and no adverse events were recorded; however, evidence on safety is very limited.

## RSV—prophylaxis for preterm infants reduces the rate of hospital re-admissions but not mortality

Respiratory syncytial virus (RSV) is a leading cause of morbidity and mortality through lower respiratory tract infections among young infants [49]. Re-hospitalization due to RSV infection affects 0.5% up to 10% of preterm infants which is mainly influenced by gestational age and season of discharge [50]. Passive immunization of preterm infants with immunoglobulins (i.e., palivizumab) has proven safety and tolerability [51]. Monthly administration during RSV season has demonstrated a relative reduction in RSV-associated hospital admissions of 55%, but no difference with regard to the length of NICU stay, duration of ventilation or mortality [51]. There is an ongoing discussion about cost-effectiveness of RSV prophylaxis in infant populations with different levels of susceptibility. A reasonable approach is to limit passive immunization to high-risk infants before discharge into RSV season (i.e., predisposition with chronic lung disease, congenital heart disease). Potential future approaches include the passive immunization with a long-lasting monoclonal antibody, i.e., nirsevimab, which may reduce medically attended RSV infections in preterm infants by 70% [52]. A phase III trial on maternal immunization aiming at transplacental IgG-transfer has demonstrated a significant reduction in hospitalization from RSV disease by 44% in the offspring [53].

## Distinct features of neonatal immunity with relevance to vaccine responses

In order to improve immunization strategies for the specific situation of preterm infants, it is important to have a comprehensive understanding of the distinct neonatal features of immunity. There are major immunological differences between preterm and term infants with relevance to vaccine responses, for example: (i) reduced amounts of protective maternal antibodies, (ii) reduced barrier function of skin and mucosa (iii) immature innate immune function (leukocyte migration ↓, complement function↓, numbers of dendritic cells and monocytes↓, diminished antigen presentation by high adenosine levels), (iv) predominance of transitional B cells with reduced antibody affinity and maturation, (v) distinct cytokine profiles of T cells upon Toll-like receptor stimulation (Interferon-γ↓, IL-10↑) and (vi) reduced proliferation of T cell effector and memory cells [3, 29]. Preterm infants primarily rely on innate immune processes and barrier protection for their defense against pathogens. It has been proposed that innate immunity is also crucial for priming the adaptive immunity; however, data in preterm infants are scarce. We hereby focus on mechanistic insights into the B cell and T cell development and function in the early life context.

The cognate interplay of B cells and CD4 + T follicular helper (T_FH_) cells within germinal centers (GCs) of secondary lymphoid organs has emerged as an essential component for the induction of effective vaccine responses [54]. Indeed, B cells receiving adequate help from T_FH_ cells differentiate into memory B cells and long-lived plasma cells that constitute the cellular basis of long-lasting protection against re-infection [55]. The outcome of antigen-triggered vaccination responses is shaped by (i) the composition of the primary immunoglobulin repertoire of naïve B cells that fuel the response, (ii) the functional properties of T helper (Th) cells as well as (iii) the chemokine milieu provided by dendritic cells that initially prime the response. Immaturity-related decreased function of these key components has long been considered as a reason for poorer vaccine responses in early life [56]. In preterm infants, responses to Toll-like receptor (TLR)-signaling seem to be partially attenuated and fail to induce Th-1 priming cytokines (e.g., IL-12, interferon- γ). The differentiation of CD4 + T helper (Th) cells is skewed toward Th-2 cells, while the generation of T_FH_ cells and GC B cell responses is hampered and antibody responses are often transient [57, 58]. Recent studies, however, prompt a reinterpretation of B and T cell function in (preterm) neonates [59, 60]. Instead of being impaired, neonatal T and B cells rather seem to be adapted for encountering distinct immune challenges present at the beginning of life. Understanding their specific functional properties may therefore guide the way to individualized and effective vaccine approaches in preterm neonates.

## Restricted B cell receptor repertoire and relaxed tolerance threshold of neonatal B cells

**Fig. 1:**
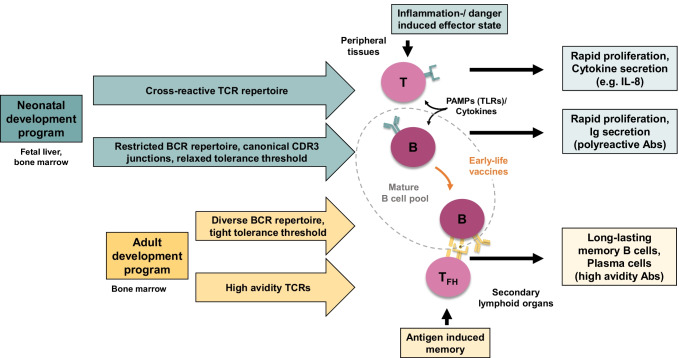
B- and T cell development. B and T cell development can be considered to proceed in different layers of intertwined development trajectories that fuel the mature cell compartment at different rates during ontogeny. The “neonatal” and adult “layer” differ in molecular characteristic and functionalities. Ideal early life vaccines could target distinct B and T cell clones displaying the benefits of a neonatal TCR/BCR repertoire and recruit these clones into affinity-maturation pathways and the long-lasting memory compartment

In mice, B cell development follows two major developmental branches. A distinct subset of B-1 cells is generated from “neonatal” precursors in the fetal liver and to a lesser extent in the bone marrow. “Conventional” B-2 cells originate from “adult” precursors in the bone marrow [61, 62]. Whereas “adult” B-2 cells participate in GC reactions, cooperate with Th cells and differentiate into memory B cells, B-1 cells rather get activated by innate stimuli (e.g., Toll-like receptor co-stimulation) and secrete semi-invariant immunoglobulin M (IgM) antibodies that are enriched in poly-reactivity and also autoreactivity [63]. Random V(D)J-recombination creates a diverse B cell repertoire (BCR) in adults, but neonatal B cells rather generate a restricted BCR that is characterized by preferential usage of distinct V- and J-gene segments and lower complementarity-determining region (CDR)3 junctional diversity due to limited incorporation of N-nucleotides [64–70]. Usage of a restricted “fetal/neonatal” versus a diverse “adult” BCR repertoire in developing B cells follows an intrinsically determined trajectory during ontogeny that does not seem to be expedited by preterm birth or other environmental influences [71–73]. Restriction of the fetal/neonatal BCR diversity has been suggested as a means of limiting the generation of autoreactive B cells. In adults, several B cell tolerance checkpoints are in place that limit further differentiation of autoreactive B cells clones generated in the bone marrow [74, 75]. Recent investigations showed that these tolerance checkpoints also exist in human fetal B cells [75]. Tolerance thresholds, however, are relaxed in early life permitting the accumulation of polyreactive clones in the mature naïve B cell pool. These polyreactive B cells by nature showed some degree of autoreactivity but also recognized commensal bacteria, a feature that was less present in the polyreactive naïve B cells from adults [75]. Neonatal naïve B cells in human fetuses seem to share characteristics with murine B-1 cells in mice, but the precise identification of a distinct B-1 cell subset in humans is still controversial [76, 77]. Therefore, neonatal (“B-1 like”) and adult (B-2 like”) cells might not be regarded as two consecutive stages of B cell development (“immature towards mature”), but rather represent two layers of intertwined B cell development trajectories that fuel the mature B cell compartment at different rates during ontogeny (Fig. 1).

## Polyreactive B cells—a meaningful role for antimicrobial responsiveness?

Active recruitment of polyreactive B cell clones into the mature naïve B cell compartment and positive selection by commensal bacteria or self-antigens is the characteristic feature of the fetal/neonatal B cell development program. There are potential risks of a relaxed B cell tolerance threshold. The molecular program licensing polyreactive/autoreactive B cell output during ontogeny, however, is probably a meaningful selection rather than a simple by-product of immaturity. In mice, this program is actively induced and under molecular control of the RNA binding protein Lin28b [72]. It is suggested, that Lin28b expression and relaxed tolerance thresholds during fetal/neonatal development permit the incorporation of distinct polyreactive specificities into the mature naïve B cell pool. This is beneficial for the newborn as the “pattern recognition” signature of polyreactive B cells convey an immediate layer of broad antimicrobial reactivity [78]. Additionally, encounters with commensal and self-antigens by polyreactive BCRs and TLRs improve the developmental fitness of these B cell clones, prime them for optimal vaccine responses and may induce a natural memory B cell compartment [79–81]. In line with this, the secondary IgG repertoire of preterm neonates is subject to similar age-related changes without preferential selection of B cell clones with an “adult” repertoire [73]. Moreover, early life immunization in mice with *S. pyogenes* induced incorporation of unique clonotypes into the memory B cell repertoire that were not stimulated by immunization of adult mice and were recruited from the B-1 cell pool [82]. Hence, early immunization at the beginning of life might open the chance to incorporate unique specificities from the “neonatal” B cell pool into the memory B cell compartment. The presence of maternal antibodies might even favor the recruitment of a broader BCR diversity by binding immunodominant epitopes and eliciting a broadly reactive repertoire with beneficial polyreactivity in the newborn [83–85]. On the other hand, vaccination in the presence of maternal antibodies does not prevent B cell activation and GC formation but impinges on the GC output by reducing plasma cell differentiation [83].

## Cross-reactive T cell receptor repertoire and “danger-signal”-induced T cell proliferation

Neonatal T cells display features that may be misinterpreted as being “deficient.” For example, the T cell receptor (TCR) repertoire of neonatal T cells is less diverse and characterized by germline-encoded TCRs that display increased cross-reactivity [67, 86–88]. These features may contribute to heterologous immunity and non-pathogen specific beneficial effects, e.g., by live vaccines. Neonatal T cells are also less responsive to stimulation by antigens but prone to receive additional signaling via TLRs and/or cytokines (e.g., IL-12 and IL-18), which led to the suggestion of and “danger responsiveness” rather than “antigen responsiveness” of these cells [89–92]. Under appropriate stimulation neonatal Th cells may show rapid proliferation and differentiation into effector cells that secrete a unique set of cytokines (e.g., IL-8) and migrate to peripheral tissues [60, 90]. However, migration into lymph nodes and differentiation into T_FH_ cells within GC reactions is hampered suggesting a differentiation bias that favors effector state over memory formation [58]. Similar to neonatal T cells, cord blood naïve B cells from neonates are also highly responsive to co-stimulation by TLR-signaling (e.g., TLR9), whereas adult naïve B cells required T cell help for optimal stimulation [93]. Also, neonatal naïve B cells are skewed to rapid proliferation and differentiation into short-lived plasma cells rather than memory B cells or long-lived plasma cells [93]. Hence, neonatal T cells share many characteristics with the peculiarities outlined for B cells at the beginning of life and rather than being immature might be intrinsically primed for responding to danger signals (TLR co-stimulation), prone to differentiate into fast-acting, short-lived effector cells and respond to a broad variety of antigens due to their polyreactive/cross-reactive repertoires (Fig. 1). Finally, preterm infants have an increased number of regulatory T cells (Tregs) which are thought to play an important role in the suppression of anti-maternal immune responses but also in dampening inflammatory responses initiated by the establishing microbiota. On the other hand, the heightened Treg responses may contribute to infection risk in preterm infants and therefore provide a cellular target for future vaccination strategies [94].

## Strategies for optimizing the vaccine protection of preterm infants

An optimal immunization for preterm infants would elicit a protective immune response following a single vaccine dose. Given the naïve immune cell status and the immunological specificities of the preterm infant, this is a consistent challenge. Herein, we discuss potential aspects toward a more personalized approach to immunization including (i) the use of novel adjuvants, (ii) promoting maternal immunization, (iii) new cellular targets (tissue-resident memory cells), (iv) new vaccines (mRNA) and (v) the influence of microbiota on vaccine responses.

## Novel adjuvants to target B and T cell developmental programs

A main challenge for early life vaccine development will be to design antigen/adjuvant formulations specifically for preterm infants. Ideally, these approaches should enable recruitment of B and T cell clones with beneficial TCR/BCR repertoire into affinity-maturation pathways and the long-lasting memory compartment. Alum adjuvants, which are traditionally used in multivalent immunizations of preterm infants, stimulate innate immune pathways via intracellular inflammasome signaling and thereby generate adaptive Th2 responses. Enhanced immunogenicity has been demonstrated by novel adjuvants such as AS01 as part of the malaria vaccine RTS,S. AS01 consists of liposomes, i.e., MPL and QS-2, which needs to be explored as adjuvant for preterm infant vaccinations [95]. It is well known that responsiveness to different adjuvants is clearly dependent on age. For example, the combination of TLR agonists and C-type lectin receptor agonists synergistically induced a shift toward Th1 polarization when used with human cord blood derived DCs but not with adult DCs [96]. Additionally, the use of cGAMP (a *stimulator of the interferon genes* (STING) ligand) plus alum as an adjuvant for influenza vaccination efficiently fostered humoral and cellular aspects of Th1 responses in newborn mice [97]. Also, co-administration of HepB vaccination with BCG vaccine induced an adult-like innate cytokine responses of cord blood mononuclear cells in vitro and significantly higher anti-HepB IgG titres in preterm mice, the latter being dependent on the application of the two vaccines at distinct injection sites [98]. These and other observations have led to the suggestion to apply the concept of precision medicine also to vaccinology, e.g., to design individualized vaccinations that are tailored to the early life setting [99].

## Protecting the infant by immunizing the mother-to-be

Maternal immunization protects against infection of the offspring by antibodies transmitted to through the placenta, which starts at the second trimester of pregnancy, or via breast milk feeding after birth. There is sufficient evidence that antenatal vaccination against tetanus, pertussis and influenza is safe and reduces disease-specific morbidity and mortality in of the offspring by up to 90% [100–104]. According to gestational age, preterm infants may have lower levels of maternal antibodies against measles and will potentially lose protection earlier than term infants [105]. Therefore, it is important to establish the molecular determinants of breast-milk transferred antibodies in preterm infants and to elucidate whether these antibodies can complement antibodies transferred through the placenta. Notably, the presence of maternal antibodies may interact with vaccine antibody responses early in life [12, 106]. Interference effects of maternal antibodies with the developing immune response of infants are observed for both priming and booster vaccine doses. Multiple mechanisms may contribute to interference effects of maternal antibodies, including epitope masking, binding of antibody to the FcγRIIB on B cells and prevention of the differentiation of GC B cells into plasma cells and memory B cells [83]. Under specific circumstances, maternal antibodies can promote immune responses in the offspring which deserves further investigation. Other areas of research to improve the neonatal immune fitness via the mother-infant dyad include insights into the role of maternal inflammation during pregnancy (e.g., chronic hepatitis) which may lead to increased antimicrobial cytokine production of neonatal cells after ex vivo stimulation with various bacterial pathogens [107]. Further, maternal immunological experience supports the programming of neonatal immunity by persisting maternal cells and protects against early-life infection (e.g., microchimerism) [108].

## Tissue-resident memory T cells—a new target for the improvement in immunization strategies?

Mechanistic studies revealed that a newly identified group of non-circulating cells including T cells (innate lymphocytes, innate-like T cells, conventional T cells), B cells and macrophages already establish during pregnancy in the fetus [109, reviewed in 110]. The term tissue-resident memory (Trm) T cells refers to populations of conventional T cells, as well CD4 + as CD8 + , that acquire tissue-resident characteristics. These lymphocytes are predominantly located in non-lymphoid tissues such as gut, skin, lung and materno-fetal interfaces, in contrast to naïve adaptive lymphocytes, which constantly circulate between secondary lymphoid organs. Some non-lymphoid organs harbor a sizable population of tissue-resident lymphocytes, including Trm T cells, unconventional T cells such as invariant natural killer T (iNKT) cells, intraepithelial lymphocytes (IEL), γδ T cells and a diverse family of innate lymphocytes [111, 112]. Stras et al. [109] observed that the mucosal adaptive immune system of the fetal gut, which is exposed to antigens from the amniotic fluids, can mount robust adaptive immune responses. Trm T cells have a specific role for protective immunity against invading microorganisms at the barrier tissue where they reside [112]. Their unique features allow them to elicit an immediate and site-specific effector response in the tissue after secondary challenge to an infectious agent [113]. In the lung, for example, the fast response of CD4 + and CD8 + Trm T cells is possible through high baseline levels of mRNA encoding inflammatory molecules such as granzyme B and cytokines, i.e., interferon-gamma [111, 113]. Trm T cells typically express CD69 which hinders them from migrating from tissues into blood and other retention markers/integrins such as CD103 and CD49a. Several studies have enlightened the role of Trm T cells for protection at mucosal sites against infections such as tuberculosis, CMV, HSV-2, EBV and viral respiratory diseases in adults [113].

## Insufficient protective immunity by tissue-resident memory T cells in infants

**Fig. 2:**
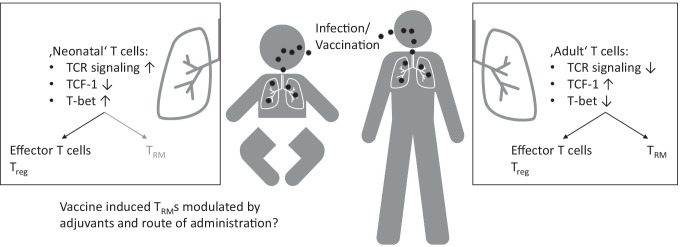
Tissue-resident memory T cells in the context of neonatal infection. Upon neonatal infection with respiratory viruses, T cells are effectively recruited to the lung. Due to the ‘neonatal’ T cell intrinsic transcriptional state, T cells are primed for an effector response. This establishes a fast and robust defense against the infection but fails to install a long-lasting protection against re-infection. The latter is achieved in adults by the formation of tissue-resident memory cells after infection or administration of vaccines

In early infancy, however, pathogen-specific T cells do not persist in a manner similar to those generated in adults. In contrast, early development of the T cell compartment favors rapid proliferation and differentiation rather than generation of long-lived tissue-resident memory. This may contribute to the increased susceptibility of young (preterm) infants to viral infections, e.g., influenza or RSV bronchiolitis, as compared to older children and adults. In an influenza mouse model, early life infection and intranasal vaccination resulted in effective mobilization of CD4 + and CD8 + T cell responses in the lung and robust viral clearance. However, the establishment of lung persistent tissue-resident memory T cells and in situ protective immunity was insufficient [114, 115]. Connors et al. [115] confirmed this data in the human context and noted the appearance of CD69 + Trms in the respiratory tract of infants as early as 6 weeks of life. Full Trm maturation including phenotypic CD103 expression (which is important for CD8 + Trm effector function) occurred after the first year of life. A potential underlying mechanism of decreased Trm generation is the increased expression of the transcriptional factor T-bet in mouse and human T cells during infancy [115]. Targeting intrinsic alterations in transcriptional regulations of T cells, i.e., modulation of T-bet expression may therefore be advantageous for promoting, long-lived Trm populations in the respiratory environment. Another example is the reduced production of IFN-α/ß in animal models of RSV infection which causes impaired expansion of CD8 + Trm cells and can be restored by treatment with IFN-α [116]. Figure 2 shows that the ‘neonatal ‘ T cell intrinsic transcriptional pattern is primed to elicit a strong effector response but fails to install a long lasting protection against re-infection which could be modulated by adjuvants or different routes of administration.

## The route of vaccine administration influences tissue-resident memory T cell responses

**Fig. 3:**
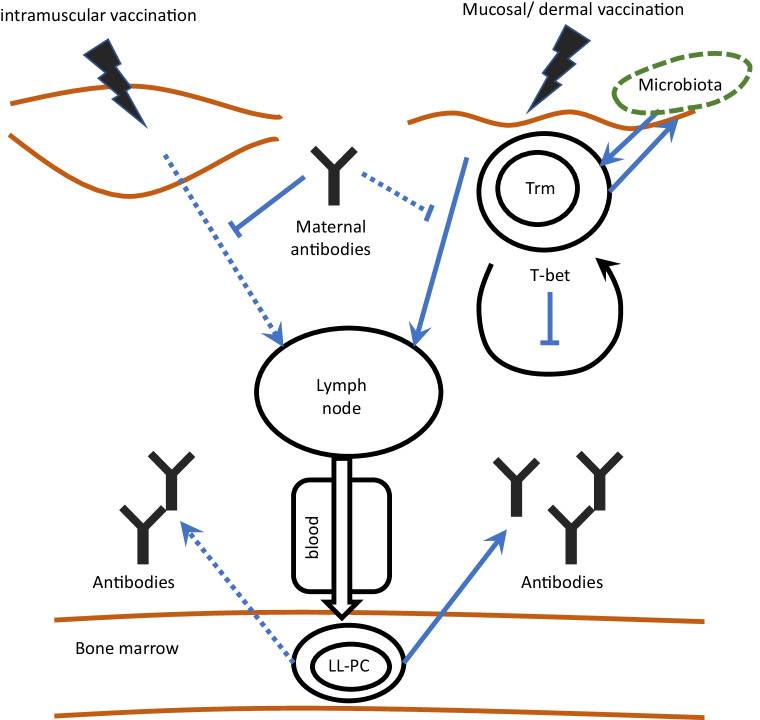
Tissue-resident immunity after intramuscular versus mucosal/dermal vaccination in neonates. Intramuscular vaccination elicits a weaker Tissue-resident immune response than mucosal vaccination due to higher interference with maternal antibodies and proposingly reduced interaction with the local microbiome. Tissue-resident memory cells, LL-PC: Long-lived plasma cell

It has been recently demonstrated that intranasal vaccination may stimulate the generation of virus-specific Trms in a more protective fashion than systemic vaccines [117, 118] and may also reduce the interference with maternal antibodies in the blood. In line with that, intranasal delivery of a ChAd-SARS-CoV2-S vaccine induced a potent, local cell-mediated immunity by CD4 + and CD8 + Trm. Intranasal vaccination was more effective in inhibiting viral replication as compared to intramuscular delivery of the vaccine [119]. Hence, future vaccinations directly administered to the mucosa may be a powerful way to strengthen the site-specific immune response in infants [117, 120]. Figure 3 shows a general schematic overview of the tissue-resident immunity after intramuscular in comparison with mucosal/dermal vaccination in early life. This also points to the putative interaction of Trm T cells with the establishing microbiota and the associated metabolites. The processes of generating microbiota-specific Trm T cells have not been thoroughly elucidated in early life immunity yet [reviewed in 121]. Given the high vulnerability of the neonatal microbiome for disturbance by environmental factors (as discussed below), the proposed function of Trm T cells to “remember” benign interactions with the microbiome as a preventive measure against repetitive or sustained inflammation [121] might also be affected. In the context of preterm birth, the lack of IgA from breast milk and maternal IgG antibodies transferred through the placenta may also result in reduced shaping of Trm T cells after birth [122].

Targeting Trm T cells might also become a new strategy to develop vaccines against congenital cytomegalovirus infection, which is a frequent cause of long-term neurodevelopmental sequelae. Infection of newborn mice with mouse cytomegalovirus (MCMV) intraperitoneally demonstrated that CD8^+^ T cells infiltrate the brain and generate a pool of long-lived CD8 + Trms. When virus-specific CD8^+^ T cells were adoptively transferred, the Trm pool provided protection against primary MCMV infection in newborn mice and reduced brain pathology, while depletion of MCMV-specific Trms resulted in virus reactivation and enhanced brain inflammation [123].

## Improving vaccine responses by modulating the microbiome

The intestinal microbiota plays a crucial rule in educating the immune system and serves as a natural source of adjuvants (antigens, metabolic products such as short-chain fatty acids) which is critical for immune responses to vaccination [124–127]. The mechanistic background has been derived from animal models demonstrating that antibiotic-treated, germ-free or knockout TLR5 mice have markedly reduced antibody production after influenza or inactivated polio vaccination as compared to conventional mice [128]. Early life treatment of mice with antibiotics also led to impaired antibody responses against five different live or attenuated licensed vaccines which was rescued by fecal microbiota transplantation from age-matched control mice [129]. In the human context, the gut microbiome abundance of *Bifidobacteria*—the champion colonizer of human-milk fed term infants—seems to support thymic development and correlates with a high magnitude of T cell responses against oral polio vaccine, BCG and tetanus vaccines in the first months of life [130]. The *Bifidobacteria*-dominated microbiome signature sustainably improves immunological memory, i.e., CD4 responses to BCG and tetanus, tetanus-specific IgG and stool polio-specific immunoglobulin A at 2 years of age [131]. Deviation from this pattern, resulting in greater bacterial diversity, may cause systemic inflammation (neutrophilia) and impaired vaccine responses [130]. Microbiota establishment is highly dynamic, variable and therefore susceptible to disturbance, particularly in preterm infants who are often exposed to risk factors of dysbiosis (i.e., Caesarean section, exposure to antibiotics, reduced options for skin-to-skin contact and lack of human milk feeding). Therefore, beneficial modulation by microbiota-targeted interventions (nutrition, prebiotics, probiotics) has been an attractive approach to optimize vaccine responses [132]. In a mouse model for infants’ undernutrition, the combined prebiotic and probiotic intervention resulted in improved antibody responses to oral cholera vaccine [133]. A recent meta-analysis summarized data of different, often small scale studies on effects of probiotic supplementation on immune responses to 17 different vaccines [134]. Positive effects on vaccine responses were seen for about 50% of the probiotic formulations; however, cautious interpretation is needed due to the large heterogeneity in study designs, cohorts and probiotic strains. Preterm infants often receive *Bifidobacteria*-containing probiotics to reduce the risk of necrotizing enterocolitis in the first weeks of life [135]. Whether these early interventions stabilize the microbiota and positively impact on vaccine responses of preterm infants needs to be further investigated.

## Novel vaccines: mRNA technology

The use of mRNA vaccines could provide an attractive option for future personalized vaccination of preterm infants. In principle, mRNA vaccines are manufactured in a cell-free manner and have several advantages over conventional vaccines, specifically: (i) no integration into the genome (unlike some viral vaccines) or concerns about insertional mutagenesis and (ii) rapid, scalable and cost-effective production and inexpensive storage in a lyophilized fashion. A single mRNA vaccine formulation can encode multiple antigens thereby eliciting an immune response against several resilient pathogens or viral variants [136] which would also enhance compliance to immunization among families and medical professionals. In the neonatal context, first insights into robust immunogenicity of mRNA vaccines were derived from a mouse model demonstrating that an influenza nucleoside-modified mRNA encapsulated in lipid nanoparticles (mRNA-LNP) partially overcame the inhibition by maternal antibodies. The mRNA-LNP influenza vaccine established long-lived GC reactions and generated stronger antibody responses than a conventional influenza vaccine [137]. In addition, mRNA vaccines can be rapidly adjusted to microbial mutations or variants of concern which may, for example, improve the design for active RSV vaccines. Initial concerns about mRNA vaccines are related to stability, poor efficacy and unknown side effects such as excessive immunostimulation. Therefore, dose finding is crucial, particularly in vulnerable infants. For example, recent data on SARS-CoV2 mRNA vaccination with Comirnaty demonstrate that dose adjustments to 30 µg have resulted in poor immunogenicity in children under 5 years [138]. Most importantly, the potential future use of mRNA vaccines in vulnerable preterm infants must be above any safety concerns [136].

## Outlook

Toward a more personalized approach for vaccination the main challenge is that currently no vaccines exist for the most frequent causes of systemic infections in preterm infants, caused by staphylococci, Group B streptococci, Escherichia coli and other Gram-negative rods. With the advance of research for new adjuvants, emerging vaccine technologies, development of new targets with specific focus on tissue-resident memory formation and a deeper insight into the delicate interplay between microbiota establishment and infant immunity there is hope for significant advances in the future.

Current protection of preterm infants relies on improved communication of the evidence on safety and efficacy of vaccinations in preterm infants and on promoting the adherence to up-to-date immunization recommendations. Timely vaccination has proven effects on risk reduction for vaccine-preventable diseases, while the benefit of pathogen-agnostic trained immunity processes in preterm infants needs to be further delineated. A balanced approach between an intended immune stimulation and uncontrolled inflammation is required for the specific situation of preterm infants. A large proportion is born prematurely due to inflammatory conditions at the materno-fetal interface (“first hit”), and preterm infants often lack capacities to counter-regulate inflammation accordingly. Given the frequent exposure to secondary hits, sustained inflammation might occur which is associated with adverse outcome [3]. Beyond non-specific measures of preventing postnatal inflammation (i.e., less intensive care, antibiotic stewardship) individualized, family-centered developmental care (i.e., stress reduction, maintaining circadian rhythms) is important. Intriguingly, neurodevelopment and immune maturation share common trajectories and mutually interact as a continuum. For example, circadian clocks control the infiltration of dendritic cells into skin lymphatics in mice which is essential for adaptive immunity and relevant for vaccination responses [139]. Sleep after vaccination results in superior immunogenicity as compared to antibody levels in adults who stay awake [140]. A small-scale study in preterm infants found no difference between morning and evening vaccination with regard to antibody titres and side effects [141], while further studies are needed to target the circadian immune response interaction in preterm infants.

To develop individualized immunizations in preterm infants, future studies can take advantage of the unique, highly controlled observational conditions of preterm infants in the neonatal unit. During a critical developmental window, most highly preterm infants receive their first vaccinations in hospital which provides the basis for large-scale cohort studies. Systems medicine and integrative modelling of host genetic, clinical data and multi-omics data can therefore help to disentangle complex interdependent relationships early in life and derive personalized risk-and-benefit patterns.

## Supplementary Information

Below is the link to the electronic supplementary material.

Supplementary file1 (DOCX 29 KB)
