# Supplementary material for: Immunization of preterm infants: current evidence and future strategies to individualized approaches
Source: Semin Immunopathol. 2022 Aug 3;44(6):767–84. doi: 10.1007/s00281-022-00957-1 (PMC9362650; doi:10.1007/s00281-022-00957-1)
Supplement: Supplementary file 1 — Supplementary file1 (DOCX 29 KB) [file 281_2022_957_MOESM1_ESM.docx]

**Supplement 1**

**SSIM D22-00017 Immunization of preterm infants: current evidence and future strategies to individualized approaches**

Mats Ingmar Fortmann^1*^, Johannes Dirks^2*^, Sybelle Goedicke-Fritz^3*^, Johannes Liese^2^, Michael Zemlin^3^, Henner Morbach^3^, Christoph Härtel^2^

**Supplement 1: References to table 1**

1. Vesikari T, Matson DO, Dennehy P, et al. Safety and efficacy of a pentavalent human-bovine (WC3) reassortant rotavirus vaccine. *N Engl J Med*. 2006;354(1):23-33. doi:10.1056/NEJMoa052664

2. Ruiz-Palacios GM, Pérez-Schael I, Velázquez FR, et al. Safety and efficacy of an attenuated vaccine against severe rotavirus gastroenteritis. *N Engl J Med*. 2006;354(1):11-22. doi:10.1056/NEJMoa052434

3. Van der Wielen M, Van Damme P. Pentavalent human-bovine (WC3) reassortant rotavirus vaccine in special populations: a review of data from the Rotavirus Efficacy and Safety Trial. *Eur J Clin Microbiol Infect Dis Off Publ Eur Soc Clin Microbiol*. 2008;27(7):495-501. doi:10.1007/s10096-008-0479-5

4. Goveia MG, Rodriguez ZM, Dallas MJ, et al. Safety and efficacy of the pentavalent human-bovine (WC3) reassortant rotavirus vaccine in healthy premature infants. *Pediatr Infect Dis J*. 2007;26(12):1099-1104. doi:10.1097/INF.0b013e31814521cb

5. Roué JM, Nowak E, Le Gal G, et al. Impact of rotavirus vaccine on premature infants. *Clin Vaccine Immunol CVI*. 2014;21(10):1404-1409. doi:10.1128/CVI.00265-14

6. Thrall S, Doll MK, Nhan C, et al. Evaluation of pentavalent rotavirus vaccination in neonatal intensive care units. *Vaccine*. 2015;33(39):5095-5102. doi:10.1016/j.vaccine.2015.08.015

7. Monk HM, Motsney AJ, Wade KC. Safety of rotavirus vaccine in the NICU. *Pediatrics*. 2014;133(6):e1555-1560. doi:10.1542/peds.2013-3504

8. Hiramatsu H, Suzuki R, Nagatani A, et al. Rotavirus Vaccination Can Be Performed Without Viral Dissemination in the Neonatal Intensive Care Unit. *J Infect Dis*. 2018;217(4):589-596. doi:10.1093/infdis/jix590

9. Iwata S, Nakata S, Ukae S, et al. Efficacy and safety of pentavalent rotavirus vaccine in Japan: a randomized, double-blind, placebo-controlled, multicenter trial. *Hum Vaccines Immunother*. 2013;9(8):1626-1633. doi:10.4161/hv.24846

10. Omenaca F, Sarlangue J, Szenborn L, et al. Safety, reactogenicity and immunogenicity of the human rotavirus vaccine in preterm European Infants: a randomized phase IIIb study. *Pediatr Infect Dis J*. 2012;31(5):487-493. doi:10.1097/INF.0b013e3182490a2c

11. Bhave S, Bhise S, Chavan SC, et al. Hepatitis B vaccination in premature and low birth weight (LBW) babies. *Indian Pediatr*. 2002;39(7):625-631.

12. Waitz M, Hopfner R, Hummler HD, Heininger U. Hepatitis B Postexposure Prophylaxis in Preterm and Low-Birth-Weight Infants. *AJP Rep*. 2015;5(1):e67-72. doi:10.1055/s-0035-1547329

13. Ellison VJ, Davis PG, Doyle LW. Adverse reactions to immunization with newer vaccines in the very preterm infant. *J Paediatr Child Health*. 2005;41(8):441-443. doi:10.1111/j.1440-1754.2005.00663.x

14. Omenaca F, Garcia-Sicilia J, García-Corbeira P, et al. Response of preterm newborns to immunization with a hexavalent diphtheria-tetanus-acellular pertussis-hepatitis B virus-inactivated polio and Haemophilus influenzae type b vaccine: first experiences and solutions to a serious and sensitive issue. *Pediatrics*. 2005;116(6):1292-1298. doi:10.1542/peds.2004-2336

15. Lau YL, Tam AY, Ng KW, et al. Response of preterm infants to hepatitis B vaccine. *J Pediatr*. 1992;121(6):962-965. doi:10.1016/s0022-3476(05)80352-x

16. Losonsky GA, Wasserman SS, Stephens I, et al. Hepatitis B vaccination of premature infants: a reassessment of current recommendations for delayed immunization. *Pediatrics*. 1999;103(2):E14. doi:10.1542/peds.103.2.e14

17. Saari TN. Immunization of preterm and low birth weight infants. American Academy of Pediatrics Committee on Infectious Diseases. *Pediatrics*. 2003;112(1 Pt 1):193-198. doi:10.1542/peds.112.1.193

18. Fan W, Zhang M, Zhu YM, Zheng YJ. Immunogenicity of Hepatitis B Vaccine in Preterm or Low Birth Weight Infants: A Meta-Analysis. *Am J Prev Med*. 2020;59(2):278-287. doi:10.1016/j.amepre.2020.03.009

19. Blondheim O, Bader D, Abend M, et al. Immunogenicity of hepatitis B vaccine in preterm infants. *Arch Dis Child Fetal Neonatal Ed*. 1998;79(3):F206-208. doi:10.1136/fn.79.3.f206

20. Saroha M, Faridi MMA, Batra P, Kaur I, Dewan DK. Immunogenicity and safety of early vs delayed BCG vaccination in moderately preterm (31-33 weeks) infants. *Hum Vaccines Immunother*. 2015;11(12):2864-2871. doi:10.1080/21645515.2015.1074361

21. Thayyil-Sudhan S, Kumar A, Singh M, Paul VK, Deorari AK. Safety and effectiveness of BCG vaccination in preterm babies. *Arch Dis Child Fetal Neonatal Ed*. 1999;81(1):F64-66. doi:10.1136/fn.81.1.f64

22. Kaur S, Faridi MMA, Agarwal KN. BCG vaccination reaction in low birth weight infants. *Indian J Med Res*. 2002;116:64-69.

23. Tun KM, Win H, Oo AK, Myint SS, Hla SK, Naing H. Tuberculin conversion after BCG vaccination: comparison by gestation and by age at immunization in Myanmar. *Trans R Soc Trop Med Hyg*. 2000;94(2):219-220. doi:10.1016/s0035-9203(00)90282-7

24. Jensen KJ, Larsen N, Biering-Sørensen S, et al. Heterologous immunological effects of early BCG vaccination in low-birth-weight infants in Guinea-Bissau: a randomized-controlled trial. *J Infect Dis*. 2015;211(6):956-967. doi:10.1093/infdis/jiu508

25. Kjærgaard J, Stensballe LG, Birk NM, et al. Lack of a Negative Effect of BCG-Vaccination on Child Psychomotor Development: Results from the Danish Calmette Study - A Randomised Clinical Trial. *PloS One*. 2016;11(4):e0154541. doi:10.1371/journal.pone.0154541

26. Kjærgaard J, Stensballe LG, Birk NM, et al. Bacillus Calmette-Guérin vaccination at birth: Effects on infant growth. A randomized clinical trial. *Early Hum Dev*. 2016;100:49-54. doi:10.1016/j.earlhumdev.2016.05.015

27. Hawkridge A, Hatherill M, Little F, et al. Efficacy of percutaneous versus intradermal BCG in the prevention of tuberculosis in South African infants: randomised trial. *BMJ*. 2008;337:a2052. doi:10.1136/bmj.a2052

28. Colditz GA, Berkey CS, Mosteller F, et al. The efficacy of bacillus Calmette-Guérin vaccination of newborns and infants in the prevention of tuberculosis: meta-analyses of the published literature. *Pediatrics*. 1995;96(1 Pt 1):29-35.

29. Faridi M, Kaur S, Krishnamurthy S, Kumari P. Tuberculin conversion and leukocyte migration inhibition test after BCG vaccination in newborn infants. *Hum Vaccin*. 2009;5(10):690-695. doi:10.4161/hv.5.10.9317

30. World Health Organization. Summary of WHO Position Papers - Recommended Routine Immunizations for Children. Published online April 2019. https://www.who.int/immunization/policy/Immunization_routine_table2.pdf?ua=1

31. Organization WH. BCG vaccine: WHO position paper, February 2018 - Recommendations. *Vaccine*. 2018;36(24):3408-3410. doi:10.1016/j.vaccine.2018.03.009

32. Biering-Sørensen S, Aaby P, Lund N, et al. Early BCG-Denmark and Neonatal Mortality Among Infants Weighing <2500 g: A Randomized Controlled Trial. *Clin Infect Dis Off Publ Infect Dis Soc Am*. 2017;65(7):1183-1190. doi:10.1093/cid/cix525

33. Biering-Sørensen S, Aaby P, Napirna BM, et al. Small randomized trial among low-birth-weight children receiving bacillus Calmette-Guérin vaccination at first health center contact. *Pediatr Infect Dis J*. 2012;31(3):306-308. doi:10.1097/INF.0b013e3182458289

34. Lee J, Robinson JL, Spady DW. Frequency of apnea, bradycardia, and desaturations following first diphtheria-tetanus-pertussis-inactivated polio-Haemophilus influenzae type B immunization in hospitalized preterm infants. *BMC Pediatr*. 2006;6:20. doi:10.1186/1471-2431-6-20

35. Clifford V, Crawford NW, Royle J, et al. Recurrent apnoea post immunisation: Informing re-immunisation policy. *Vaccine*. 2011;29(34):5681-5687. doi:10.1016/j.vaccine.2011.06.005

36. Schulzke S, Heininger U, Lücking-Famira M, Fahnenstich H. Apnoea and bradycardia in preterm infants following immunisation with pentavalent or hexavalent vaccines. *Eur J Pediatr*. 2005;164(7):432-435. doi:10.1007/s00431-005-1674-3

37. Flatz-Jequier A, Posfay-Barbe KM, Pfister RE, Siegrist CA. Recurrence of cardiorespiratory events following repeat DTaP-based combined immunization in very low birth weight premature infants. *J Pediatr*. 2008;153(3):429-431. doi:10.1016/j.jpeds.2008.03.043

38. Carbone T, McEntire B, Kissin D, et al. Absence of an increase in cardiorespiratory events after diphtheria-tetanus-acellular pertussis immunization in preterm infants: a randomized, multicenter study. *Pediatrics*. 2008;121(5):e1085-1090. doi:10.1542/peds.2007-2059

39. Pfister RE, Aeschbach V, Niksic-Stuber V, Martin BC, Siegrist CA. Safety of DTaP-based combined immunization in very-low-birth-weight premature infants: frequent but mostly benign cardiorespiratory events. *J Pediatr*. 2004;145(1):58-66. doi:10.1016/j.jpeds.2004.04.006

40. Hacking DF, Davis PG, Wong E, Wheeler K, McVernon J. Frequency of respiratory deterioration after immunisation in preterm infants. *J Paediatr Child Health*. 2010;46(12):742-748. doi:10.1111/j.1440-1754.2010.01832.x

41. Furck AK, Richter JW, Kattner E. Very low birth weight infants have only few adverse events after timely immunization. *J Perinatol Off J Calif Perinat Assoc*. 2010;30(2):118-121. doi:10.1038/jp.2009.112

42. Pourcyrous M, Korones SB, Crouse D, Bada HS. Interleukin-6, C-reactive protein, and abnormal cardiorespiratory responses to immunization in premature infants. *Pediatrics*. 1998;101(3):E3. doi:10.1542/peds.101.3.e3

43. Wilińska M, Warakomska M, Głuszczak-Idziakowska E, Jackowska T. Risk factors for adverse events after vaccinations performed during the initial hospitalization of infants born prematurely. *Dev Period Med*. 2016;20(4):296-305.

44. Ben Jmaa W, Hernández AI, Sutherland MR, et al. Cardio-respiratory Events and Inflammatory Response After Primary Immunization in Preterm Infants < 32 Weeks Gestational Age: A Randomized Controlled Study. *Pediatr Infect Dis J*. 2017;36(10):988-994. doi:10.1097/INF.0000000000001647

45. Vázquez L, Garcia F, Rüttimann R, Coconier G, Jacquet JM, Schuerman L. Immunogenicity and reactogenicity of DTPa-HBV-IPV/Hib vaccine as primary and booster vaccination in low-birth-weight premature infants. *Acta Paediatr Oslo Nor 1992*. 2008;97(9):1243-1249. doi:10.1111/j.1651-2227.2008.00884.x

46. Faldella G, Alessandroni R, Magini GM, et al. The preterm infant’s antibody response to a combined diphtheria, tetanus, acellular pertussis and hepatitis B vaccine. *Vaccine*. 1998;16(17):1646-1649. doi:10.1016/s0264-410x(98)00060-7

47. Slack MH, Schapira D, Thwaites RJ, et al. Acellular pertussis vaccine given by accelerated schedule: response of preterm infants. *Arch Dis Child Fetal Neonatal Ed*. 2004;89(1):F57-60. doi:10.1136/fn.89.1.F57

48. Schloesser RL, Fischer D, Otto W, Rettwitz-Volk W, Herden P, Zielen S. Safety and immunogenicity of an acellular pertussis vaccine in premature infants. *Pediatrics*. 1999;103(5):e60. doi:10.1542/peds.103.5.e60

49. D’Angio CT, Maniscalco WM, Pichichero ME. Immunologic response of extremely premature infants to tetanus, Haemophilus influenzae, and polio immunizations. *Pediatrics*. 1995;96(1 Pt 1):18-22.

50. Khalak R, Pichichero ME, D’Angio CT. Three-year follow-up of vaccine response in extremely preterm infants. *Pediatrics*. 1998;101(4 Pt 1):597-603. doi:10.1542/peds.101.4.597

51. Kirmani KI, Lofthus G, Pichichero ME, Voloshen T, D’Angio CT. Seven-year follow-up of vaccine response in extremely premature infants. *Pediatrics*. 2002;109(3):498-504. doi:10.1542/peds.109.3.498

52. Slack MH, Schapira D, Thwaites RJ, et al. Immune response of premature infants to meningococcal serogroup C and combined diphtheria-tetanus toxoids-acellular pertussis-Haemophilus influenzae type b conjugate vaccines. *J Infect Dis*. 2001;184(12):1617-1620. doi:10.1086/324666

53. Baxter D, Ghebrehewet S, Welfare W, Ding DCD. Vaccinating premature infants in a Special Care Baby Unit in the UK: results of a prospective, non-inferiority based, pragmatic case series study. *Hum Vaccin*. 2010;6(6):512-520. doi:10.4161/hv.6.6.11448

54. Heath PT, Booy R, Griffiths H, et al. Clinical and immunological risk factors associated with Haemophilus influenzae type b conjugate vaccine failure in childhood. *Clin Infect Dis Off Publ Infect Dis Soc Am*. 2000;31(4):973-980. doi:10.1086/318132

55. Kim SC, Chung EK, Hodinka RL, et al. Immunogenicity of hepatitis B vaccine in preterm infants. *Pediatrics*. 1997;99(4):534-536. doi:10.1542/peds.99.4.534

56. Kent A, Ladhani SN, Andrews NJ, et al. Schedules for Pneumococcal Vaccination of Preterm Infants: An RCT. *Pediatrics*. 2016;138(3). doi:10.1542/peds.2015-3945

57. Omeñaca F, Merino JM, Tejedor JC, et al. Immunization of preterm infants with 10-valent pneumococcal conjugate vaccine. *Pediatrics*. 2011;128(2):e290-298. doi:10.1542/peds.2010-1184

58. Esposito S, Pugni L, Bosis S, et al. Immunogenicity, safety and tolerability of heptavalent pneumococcal conjugate vaccine administered at 3, 5 and 11 months post-natally to pre- and full-term infants. *Vaccine*. 2005;23(14):1703-1708. doi:10.1016/j.vaccine.2004.09.029

59. Shinefield H, Black S, Ray P, Fireman B, Schwalbe J, Lewis E. Efficacy, immunogenicity and safety of heptavalent pneumococcal conjugate vaccine in low birth weight and preterm infants. *Pediatr Infect Dis J*. 2002;21(3):182-186. doi:10.1097/00006454-200203000-00003

60. Black S, Shinefield H. Safety and efficacy of the seven-valent pneumococcal conjugate vaccine: evidence from Northern California. *Eur J Pediatr*. 2002;161 Suppl 2:S127-131. doi:10.1007/s00431-002-1064-z

61. Moss SJ, Fenton AC, Toomey JA, Grainger AJ, Smith J, Gennery AR. Responses to a conjugate pneumococcal vaccine in preterm infants immunized at 2, 3, and 4 months of age. *Clin Vaccine Immunol CVI*. 2010;17(11):1810-1816. doi:10.1128/CVI.00214-10

62. Szynczewska E, Chlebna-Sokół D. Immunogenicity of heptavalent conjugate vaccine against Streptococcus pneumoniae in premature babies with low birth weight. *Pediatr Neonatol*. 2014;55(2):101-107. doi:10.1016/j.pedneo.2013.06.005

63. Martinón-Torres F, Czajka H, Center KJ, et al. 13-valent pneumococcal conjugate vaccine (PCV13) in preterm versus term infants. *Pediatrics*. 2015;135(4):e876-886. doi:10.1542/peds.2014-2941

64. D’Angio CT, Heyne RJ, O’Shea TM, et al. Heptavalent pneumococcal conjugate vaccine immunogenicity in very-low-birth-weight, premature infants. *Pediatr Infect Dis J*. 2010;29(7):600-606. doi:10.1097/INF.0b013e3181d264a6

65. Rückinger S, van der Linden M, von Kries R. Effect of heptavalent pneumococcal conjugate vaccination on invasive pneumococcal disease in preterm born infants. *BMC Infect Dis*. 2010;10:12. doi:10.1186/1471-2334-10-12

66. Esposito S, Corbellini B, Bosis S, et al. Immunogenicity, safety and tolerability of meningococcal C CRM197 conjugate vaccine administered 3, 5 and 11 months post-natally to pre- and full-term infants. *Vaccine*. 2007;25(26):4889-4894. doi:10.1016/j.vaccine.2007.04.018

67. Kent A, Beebeejaun K, Braccio S, et al. Safety of meningococcal group B vaccination in hospitalised premature infants. *Arch Dis Child Fetal Neonatal Ed*. 2019;104(2):F171-F175. doi:10.1136/archdischild-2017-314152

68. Collins CL, Ruggeberg JU, Balfour G, et al. Immunogenicity and immunologic memory of meningococcal C conjugate vaccine in premature infants. *Pediatr Infect Dis J*. 2005;24(11):966-968. doi:10.1097/01.inf.0000187027.45918.6a

69. Sáez-Llorens X, Castaño E, Null D, et al. Safety and pharmacokinetics of an intramuscular humanized monoclonal antibody to respiratory syncytial virus in premature infants and infants with bronchopulmonary dysplasia. The MEDI-493 Study Group. *Pediatr Infect Dis J*. 1998;17(9):787-791. doi:10.1097/00006454-199809000-00007

70. Palivizumab, a humanized respiratory syncytial virus monoclonal antibody, reduces hospitalization from respiratory syncytial virus infection in high-risk infants. The IMpact-RSV Study Group. *Pediatrics*. 1998;102(3 Pt 1):531-537.

71. Groothuis JR. Safety and tolerance of palivizumab administration in a large Northern Hemisphere trial. Northern Hemisphere Expanded Access Study Group. *Pediatr Infect Dis J*. 2001;20(6):628-630. doi:10.1097/00006454-200106000-00018

72. Subramanian KN, Weisman LE, Rhodes T, et al. Safety, tolerance and pharmacokinetics of a humanized monoclonal antibody to respiratory syncytial virus in premature infants and infants with bronchopulmonary dysplasia. MEDI-493 Study Group. *Pediatr Infect Dis J*. 1998;17(2):110-115. doi:10.1097/00006454-199802000-00006

73. Mitchell I, Tough S, Gillis L, Majaesic C. Beyond randomized controlled trials: a “real life” experience of respiratory syncytial virus infection prevention in infancy with and without palivizumab. *Pediatr Pulmonol*. 2006;41(12):1167-1174. doi:10.1002/ppul.20507

74. Singleton RJ, Bruden D, Bulkow LR, Varney G, Butler JC. Decline in respiratory syncytial virus hospitalizations in a region with high hospitalization rates and prolonged season. *Pediatr Infect Dis J*. 2006;25(12):1116-1122. doi:10.1097/01.inf.0000245104.26996.57

75. Groothuis JR, Levin MJ, Rabalais GP, Meiklejohn G, Lauer BA. Immunization of high-risk infants younger than 18 months of age with split-product influenza vaccine. *Pediatrics*. 1991;87(6):823-828.

76. D’Angio CT, Heyne RJ, Duara S, et al. Immunogenicity of trivalent influenza vaccine in extremely low-birth-weight, premature versus term infants. *Pediatr Infect Dis J*. 2011;30(7):570-574. doi:10.1097/INF.0b013e31820c1fdf

77. Groothuis JR, Levin MJ, Lehr MV, Weston JA, Hayward AR. Immune response to split-product influenza vaccine in preterm and full-term young children. *Vaccine*. 1992;10(4):221-225. doi:10.1016/0264-410x(92)90156-e

78. D’Angio CT, Wyman CP, Misra RS, et al. Plasma cell and serum antibody responses to influenza vaccine in preterm and full-term infants. *Vaccine*. 2017;35(38):5163-5171. doi:10.1016/j.vaccine.2017.07.115

79. Sasaki Y, Kusuhara K, Saito M, et al. Serum immunoglobulin levels do not affect antibody responses to influenza HA vaccine in preterm infants. *Vaccine*. 2006;24(12):2208-2212. doi:10.1016/j.vaccine.2005.11.001

80. World Health Organization. https://cdn.who.int. Table 2: Summary of WHO Position Papers - Recommended Routine Immunizations for Children. Published November 2021. Accessed January 19, 2022. https://cdn.who.int/media/docs/default-source/immunization/immunization_schedules/immunization-routine-table2.pdf?sfvrsn=3e27ab48_9&download=true

81. Australian Government – Department of Health. The Australian Immunisation Handbook. Vaccination for preterm infants. Published June 3, 2020. Accessed January 19, 2022. https://immunisationhandbook.health.gov.au/vaccination-for-special-risk-groups/vaccination-for-preterm-infants

82. Government of Canada – Department of Health. Immunization of infants born prematurely: Canadian Immunization Guide. Published December 2, 2020. Accessed January 19, 2022. https://www.canada.ca/en/public-health/services/publications/healthy-living/canadian-immunization-guide-part-3-vaccination-specific-populations/page-5-immunization-infants-born-prematurely.html

83. American Academy of Pediatrics. American Academy of Pediatrics (AAP). American Academy of Pediatrics. Published March 8, 2021. Accessed January 19, 2022. https://www.aap.org

84. Updated guidance for palivizumab prophylaxis among infants and young children at increased risk of hospitalization for respiratory syncytial virus infection. *Pediatrics*. 2014;134(2):415-420. doi:10.1542/peds.2014-1665
